# Maternal Vitamin D Status and Its Related Factors in Pregnant Women in Bangkok, Thailand

**DOI:** 10.1371/journal.pone.0131126

**Published:** 2015-07-06

**Authors:** Busadee Pratumvinit, Preechaya Wongkrajang, Tuangsit Wataganara, Sithikan Hanyongyuth, Akarin Nimmannit, Somruedee Chatsiricharoenkul, Kotchamol Manonukul, Kanit Reesukumal

**Affiliations:** 1 Department of Clinical Pathology, Faculty of Medicine, Siriraj Hospital, Mahidol University, Bangkok, Thailand; 2 Division of Maternal–Fetal Medicine, Department of Obstetrics and Gynecology, Faculty of Medicine Siriraj Hospital, Mahidol University, Bangkok, Thailand; 3 Office for Research and Development, Faculty of Medicine, Siriraj Hospital, Mahidol University, Bangkok, Thailand; 4 Department of Pharmacology, Faculty of Medicine, Siriraj Hospital, Mahidol University, Bangkok, Thailand; IPO, Portuguese Institute of Oncology of Porto, PORTUGAL

## Abstract

**Background:**

There are few data focusing on the prevalence of vitamin D deficiency in tropical countries.

**Objectives:**

We determined the vitamin D status in pregnant women and examined the factors associated with vitamin D deficiency.

**Design and Methods:**

A cross-sectional study of 147 pregnant Thai women aged 18–45 years at Siriraj Hospital (a university hospital in Bangkok, Thailand) was undertaken. Clinical data and plasma levels of 25-hydroxyvitamin D [25(OH)D], intact parathyroid hormone (iPTH), calcium, albumin, phosphate and magnesium were obtained in pregnant women at delivery.

**Results:**

The prevalence of hypovitaminosis D [defined as 25(OH)D <75 nmol/L] in pregnant women at delivery was 75.5% (95% confidence interval (CI), 67.7–82.2%). Of these, vitamin D insufficiency [defined as 25(OH)D 50–74.9 nmol/L] was found in 41.5% (95% CI, 33.4–49.9%) and vitamin D deficiency [25(OH)D <50 nmol/L] was found in 34.0% (95% CI, 26.4–42.3%) of women. The mean 25(OH)D concentration was 61.6±19.3 nmol/L. The correlation between 25(OH)D and iPTH was weak (*r* = –0.29, *P*<0.01). Factors associated with vitamin D deficiency by multiple logistic regression were: pre-pregnancy body mass index (BMI in kg/m^2^, odds ratio (OR), 0.88, 95% CI 0.80–0.97, *P* = 0.01) and season of blood collection (winter *vs*. rainy, OR, 2.62, 95% CI 1.18–5.85, *P* = 0.02).

**Conclusions:**

Vitamin D deficiency is common among pregnant Thai women. The prevalence of vitamin D deficiency increased in women who had a lower pre-pregnancy BMI and whose blood was collected in the winter. Vitamin D supplementation may need to be implemented as routine antenatal care.

## Introduction

Vitamin D is an important nutritional factor in the health of the mother and her infant. Vitamin D regulates expression of >1000 genes in humans, and vitamin D receptors are found in several tissues/cells in the human body[1]. Maternal hypovitaminosis D can affect not only the mother but also the offspring. Low levels of vitamin D in the mother have been shown to be associated with preeclampsia[2–5], gestational diabetes mellitus[6–9], postpartum depression[10, 11], preterm birth[12, 13], and small-for-gestational age[14, 15]. Insufficiency of vitamin D *in utero* or in early life has been associated with several illnesses, such as an increased risk of childhood wheezing[16], respiratory infection[17–19], type 1 diabetes mellitus[20], multiple sclerosis[21], and schizophrenia[22]. Thus, acquisition of an optimal status of vitamin D in pregnancy is crucial.

Hypovitaminosis D is not expected in a tropical country such as Thailand, where there is abundant sunshine throughout the year. Increasing urbanization that precludes outdoor exposure to the sun and elevating levels of air pollution can cause a low status of vitamin D[23]. Air pollution containing ozone absorbs ultraviolet-B photons and results in a reduction of cutaneous photosynthesis of precholecalciferol[24]. In Thai culture, women prefer to have light-coloured skin, so they usually avoid direct exposure to sunlight to prevent a tanning effect[25]. In addition, few foods naturally contain vitamin D[26]. Fortification of foods with vitamin D has not been implemented, and vitamin D supplements are not prescribed routinely, for pregnant women in Thailand.

Maternal hypovitaminosis D, as measured by circulating levels of 25-hydroxyvitamin D [25(OH)D], which is the marker of choice to represent vitamin D status[27], is common during pregnancy. Maternal hypovitaminosis D can vary according to ethnicity, geographic location, and customs in relation to local clothing[28–36]. There are few reports of the prevalence of hypovitaminosis D in pregnant women living in Southeast Asia[37–40]. A recent study revealed an extremely high prevalence of hypovitaminosis D of 83% (defined as a 25(OH)D level <75 nmol/L) in pregnant Thai women in the first trimester, and the lowest concentration of 25(OH)D was found to be in the rainy season[38].

The optimal regimen and cost-effectiveness of vitamin D supplementation during pregnancy is not known[41–43]. There are changes in vitamin D status during the course of pregnancy. Previous studies showed that maternal vitamin D status closest to delivery date were more significantly associated with adverse pregnancy outcomes such as preterm birth[44], and preeclampsia[45]. Observational studies performed at delivery found a strong relationship between vitamin D deficiency and pregnancy outcomes which were preeclampsia (odds ratio, OR = 24.7)[5] and gestational diabetes (OR = 30.8)[8]. In this study, we assessed the vitamin D status in pregnant women at delivery in Bangkok, which is located in the central region of Thailand, as well as the factors associated with a deficiency of vitamin D.

## Materials and Methods

### Patients and anthropometric data

The study protocol was approved by the Ethics Review Board of Siriraj Hospital (Mahidol University, Bangkok, Thailand), approval number Si 279/2011. Written informed consent was obtained from all subjects. Pregnant women were enrolled consecutively into the study at the time of delivery at Siriraj Hospital (Mahidol University, Bangkok, Thailand; latitude 13.45N). This hospital is a tertiary medical centre that serves majority of patients from low to middle socioeconomic status.

Pregnant women aged 18–45 years carrying a singleton fetus were included in the present study. Exclusion criteria were: chronic liver diseases; chronic kidney diseases; previous gastrointestinal surgery; pulmonary tuberculosis; lymphoma; primary hyperparathyroidism; hyperthyroidism; intake within the last 6 months of medications that could interfere with the metabolism of 25(OH)D (for example, glucocorticoids, anticonvulsants).

Eligible women were recruited during September 2011 and January 2012. Women were asked a series of questions regarding their socio-demographic data, obstetric history, use of vitamin D supplementation, and extent of exposure to sunlight. The extent of exposure to sunlight was obtained by two questions. The first question asked if any body part(s) were exposed to sunlight (without protection) between 10 am to 3 pm (the time recommended to obtain adequate exposure to sunlight)[46]. The second question focused on the estimated duration of exposure to sunlight per week. Body surface area (BSA) was calculated using Lund and Browder charts[47].

The “rainy season” was considered to last from September to October, whereas the “winter season” was from November to January. Blood was collected from pregnant women in both seasons. Pregnancy and neonatal outcomes were obtained from medical records, as was height. Weight was measured to the nearest 0.1 kg using WB-3000 Digital Weighing Scales (Tanita, Tokyo, Japan) while women were wearing lightweight clothing. The body mass index (BMI) was calculated after obtaining measurements for height and weight. For infants, height was measured using a non-elastic tape, and weight was measured using 727 Digital Weighing Scales (Seca, Hamburg, Germany) while naked.

One study focusing on vitamin D deficiency[48] in pregnant women from Mysore in India (latitude 1230N), which is on a similar latitude to Bangkok, revealed a prevalence of 66% when using a cutoff point for 25(OH)D of <50 nmol/L. Using an estimated prevalence in pregnant Thai women of 66% and a margin of error of 8% with a 95% confidence interval (95% CI) meant that a cohort of 140 pregnant women was required.

### Biochemical analyses

Levels of intact parathyroid hormone (iPTH) and total 25(OH)D were measured using an electrochemiluminescence immunoassay on an Elecsys 2010 Analyzer (Roche Diagnostics, Mannheim, Germany). Plasma levels of calcium, phosphorus, magnesium and albumin were measured using an Automated Analyzer (Modular P800; Roche Diagnostics). The intra-assay coefficient of variation (CV) for 25(OH)D was 6.9% at a mean level of 38.5 nmol/L and 5.4% at a mean level of 71.8 nmol/L. The inter-assay CV for 25(OH)D was 7.07% at a mean level of 40.3 nmol/L and 4.63% at a mean level of 84.5 nmol/L. Assay sensitivity was 7.5 nmol/L. The CV was 2.28–2.30% for iPTH, and 0.7–3.1% for albumin and cations. Total 25(OH)D was reported in nmol/L. The conversion factor to conventional units (ng/mL) was 1 nmol/L × 0.4.

### Statistical analyses

Analyses were carried out using PASW v18.0 (IBM, Armonk, NY, USA) and Microsoft Excel 2007 (Microsoft, Redmond, WA). *P* = 0.05 (two-sided) was considered significant. Continuous variables are expressed as the mean ± SD or median (interquartile range (IQR)). Categorical variables are expressed as proportions. The relationship between concentrations of iPTH and 25(OH)D was assessed using Pearson’s correlation coefficient.

For comparison of mean values among groups, we used the unpaired Student’s *t*-test for normally distributed continuous data and the Mann–Whitney U test for skewed data. The Chi-square test was used to compare categorical variables. A univariate logistic regression model was employed to estimate the crude odds ratio (OR) with 95% CI for vitamin D deficiency using the 25(OH)D level as a dichotomous categorical variable and covariates as independent variables. Variables with *P* <0.2 without multicollinearity from univariate analyses were included in multiple logistic regression analyses for factors associated with vitamin D deficiency.

Consensus on the serum level of vitamin D that reflects the optimal status of vitamin D in pregnancy is lacking. Therefore, according to the 25(OH)D level (in nmol/L), the following definitions for vitamin D status were used: vitamin D sufficiency, ≥75; hypovitaminosis D, <75; vitamin D insufficiency, 50–74.9; vitamin D deficiency, <50 [41, 49]. We used a cutoff value of 25(OH)D concentration of 50 nmol/L to assess the factors associated with vitamin D deficiency.

## Results

### Characteristics of subjects

One-hundred and forty-seven pregnant women (mean age, 28.9±6.4 (range, 18–44) years) were included. Self-reported ethnicities of participants were Thai (84.4%) and Thai–Chinese (15.6%). A total of 118 women (80.3%) were employed, 26 (17.7%) women were unemployed, and 3 (2.0%) were students. Sixty (40.8%) were nulliparous women whereas 87 (59.2%) were multiparous. A total of 68.7% of women had a monthly household income <20,000 THB (equivalent to US $667) and 36 (24.5%) had obtained an associate degree or higher.

The mean BMI before pregnancy was 22.8±5.3 (range, 14.5–43.1) kg/m^2^ and 106 (72.1%) had a mean BMI before pregnancy of <25 kg/m^2^. Difference in mean body weight during pregnancy was 14.7±5.9 kg. A total of 91.8% of women delivered at term (gestation of ≥37 weeks). Fifteen women (10.2%) were taking vitamins containing vitamin D prenatally. Median (IQR) duration of exposure to sunlight per week was 2 (range, 1–4) h. Seventy-three patients (49.7%) were enrolled during the rainy season and 74 (50.3%) were enrolled during the winter.

### Levels of vitamin D and factors associated with vitamin D deficiency

**Fig 1:**
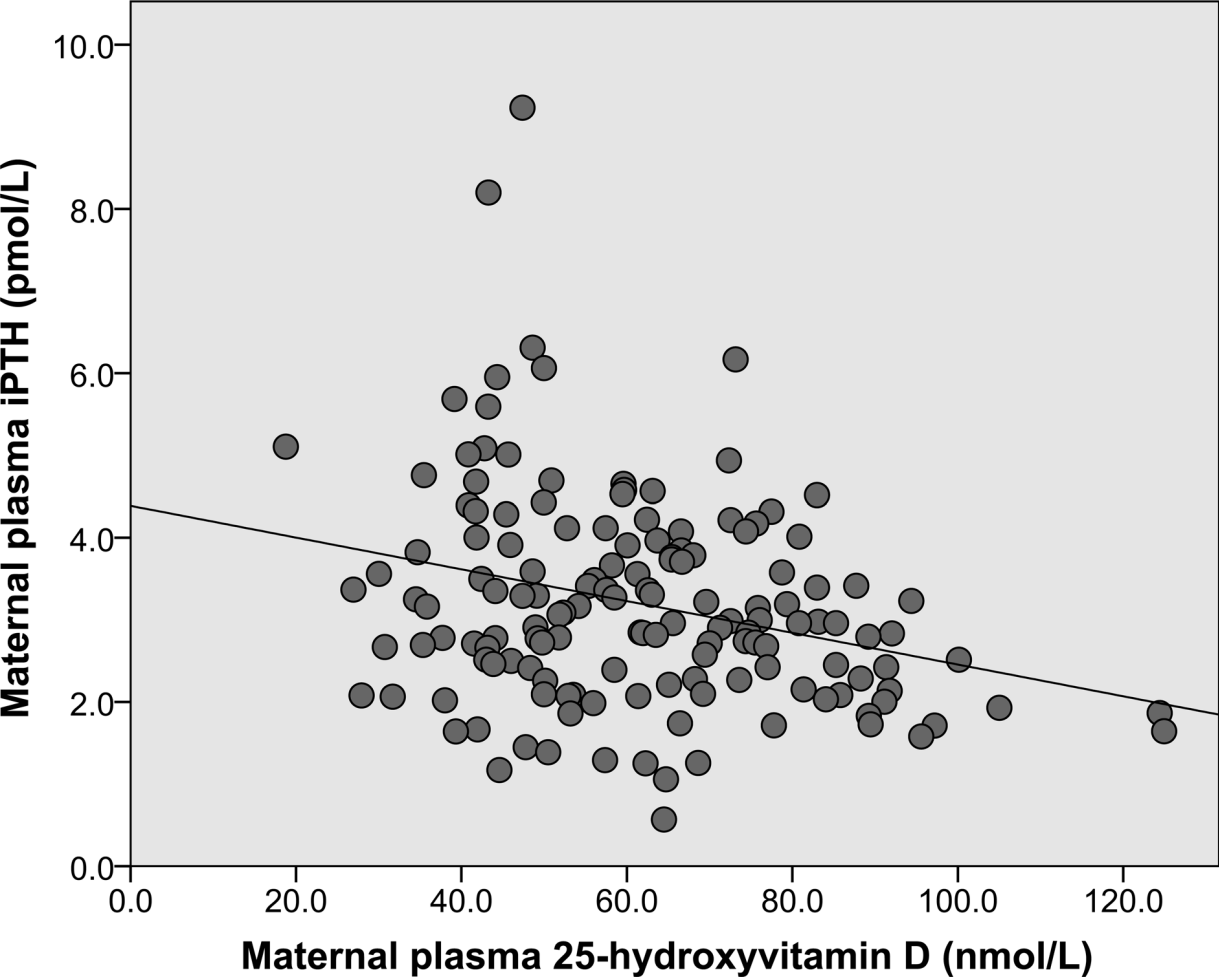
Scatter plot showing the relationship between maternal intact parathyroid hormone and 25-hydroxyvitamin D level. The maternal plasma level of 25-hydroxyvitamin D [25(OH)D]showed a weakly negative correlation with the maternal plasma level of intact parathyroid hormone (iPTH), n = 147. Linear regression equation: iPTH = –0.0193[25(OH)D] + 4.3884. (Pearson’s *r* = –0.29, *P*<0.01)

The mean concentration of 25(OH)D was 61.6±19.3 (range, 18.8–124.9) nmol/L. Thirty-six (24.5%; 95% CI, 17.8–32.3%) women had vitamin D sufficiency (25(OH)D ≥75 nmol/L). Sixty-one (41.5%; 95% CI, 33.4–49.9%) women had vitamin D insufficiency (25(OH)D 50–74.9 nmol/L), and 50 (34.0%; 95% CI, 26.4–42.3%) women had vitamin D deficiency (25(OH) D <50 nmol/L). One woman had 25(OH)D <25 nmol/L. The median (IQR) level of iPTH was 2.98 (2.27–3.91) pmol/L. An elevated level of iPTH (>6.89 pmol/L) was found in 4% (2/50) of women with vitamin D deficiency, but not in subjects with 25(OH)D ≥50 nmol/L. The maternal plasma level of 25(OH)D showed a weakly negative correlation with the maternal plasma level of iPTH (*r* = –0.29, *P*<0.01) (Fig 1).

The mean concentration of 25(OH)D showed a negative association with socioeconomic status and education level. The concentration of 25(OH)D was higher in low-educated women (secondary school or lower) than in high-educated women (associate degree or higher) (63.9±19.4 *vs*. 54.5±17.5 nmol/L, *P* = 0.01). The mean level of 25(OH)D in women with a low household income (20,000 THB) was higher than that in women with a high household income (64.1±19.3 *vs*. 56.1±18.5 nmol/L, *P* = 0.02). The proportion of high-educated women who received vitamin D supplementation was not significantly different from that in the low-educated group (11.1% *vs*. 9.9%, *P* = 0.84). Also, the proportion of women with a high household income who received vitamin D supplementation was not significantly different from that in the low-household-income group (13.0% *vs*. 8.9%, *P* = 0.44). The median (IQR) value of the product of BSA and duration of sunlight exposure per week (% BSA × h/week) in high-educated subjects tended to be lower than that in low-educated subjects but was not significantly different [51.6 (9.5–88.5) *vs*. 54.3 (26.0–113.0) m^2^h, *P* = 0.19]. The median product of BSA and duration of sunlight exposure per week for a higher household income (>20,000 THB) was lower than that for a low household income but was not significantly different [44.3 (14.4–103.3) *vs*. 54.3 (16.4–105.9) m^2^h, *P* = 0.33].

The prevalence of vitamin D deficiency was lower in subjects enrolled during the rainy season (17/73, 23.3%) than that during the winter (33/74, 44.6%). The mean value of 25(OH)D between seasons was not significantly different (63.9±17.3 in the rainy season *vs*. 59.2±21.0 nmol/L in the winter, *P* = 0.14) (Table 1). In overweight women (pre-pregnancy BMI ≥25 kg/m^2^), the prevalence of vitamin D deficiency was lower than that in women with a pre-pregnancy BMI <25 kg/m^2^ (8/41, 19.5% *vs*. 42/106, 39.6%). The mean concentration of 25(OH)D was significantly higher in pre-pregnancy overweight women compared with women with a pre-pregnancy BMI <25 kg/m^2^ (68.1±18.2 *vs*. 59.0±19.2 nmol/L, *P* = 0.01).

**Table 1:** Proportion of pregnancy with vitamin D deficiency, insufficiency, and sufficiency according to season (n = 147).

Vitamin D status (25-hydroxyvitamin D level)	Season			
	Rainy^a^		Winter^b^	
	number	%	number	%
Deficiency (<50 nmol/L)	17	23.3	33	44.6
Insufficiency (50–74.9 nmol/L)	34	46.6	27	36.5
Sufficiency (≥75 nmol/L)	22	30.1	14	18.9
**Total**	73	100	74	100

^a^Subjects recruited during September-October

^b^Subjects recruited during November-January

When using univariate logistic regression analyses, vitamin D-deficient women had higher education qualifications, higher household income, lower pre-pregnancy weight, lower pre-pregnancy BMI, higher weight gain during pregnancy, and higher increases in the BMI during pregnancy. Higher proportion of pregnant women became deficient in vitamin D during the winter. Vitamin D-deficient women had a higher mean level of iPTH (3.70±1.65 *vs*. 2.94±1.01 pmol/L, *P*<0.01) (Table 2). In multivariate analyses, pre-pregnancy BMI and season were significantly associated with the 25(OH)D level (Table 3).

**Table 2:** Univariate analysis of investigated factors associated with a plasma level of 25-hydroxyvitamin D <50 nmol/L (n = 147).

	Woman with 25(OH)D<50 nmol/L		Woman with 25(OH)D<50 nmol/L		*P-value*	Crude OR (95% CI)
	n	Mean ± SD or number (%)	n	Mean ± SD or number (%)		
Age (years)	50	29.43(6.14)	97	28.68 (6.53)	0.50	1.02 (0.97, 1.08)
Qualification, associate degree or higher	50	18 (36.0%)	97	18 (18.6%)	0.02	2.47 (1.14, 5.34)
Household income	50		97			
≤20000 THB		27 (54.0%)		74 (76.3%)	-	1
>20000 THB		23 (46.0%)		23 (23.7%)	0.01	2.74 (1.33, 5.67)
Height (cm)	50	157.84±5.08	97	157.96± 6.79	0.91	1.00 (0.94, 1.05)
Current weight (kg)	50	69.00±11.87	97	72.95±15.06	0.11	0.98 (0.95, 1.01)
Current BMI (kg/m^2^)	50	27.64±4.29	97	29.27±5.93	0.09	0.94 (0.88, 1.01)
Pre-pregnancy weight (kg)	50	52.53±9.78	97	59.20 ±14.54	0.01	0.96 (0.93, 0.99)
Pre-pregnancy BMI (kg/m^2^)	50	21.05±3.63	97	23.75±5.78	0.01	0.89 (0.82, 0.96)
Weight gain during pregnancy (kg)	50	16.47±4.96	97	13.75±6.13	0.01	1.09 (1.02, 1.17)
BMI gain during pregnancy (kg/m^2^)	50	6.59±1.89	97	5.51±2.45	0.01	1.25 (1.06, 1.48)
Nulliparity	50	25 (50%)	97	35 (36.1%)	0.11	1.77 (0.89, 3.54)
Receive vitamin-D supplement	50	6 (12%)	97	9 (9.3%)	0.61	1.33 (0.45, 3.98)
Sunlight exposure						
% BSA exposed x h/wk	49	103.0±187	97	118.3±172	0.62	1.00 (1.00, 1.00)
Hours/wk	50	3.7±5	97	4.7±7	0.39	0.98 (0.92, 1.03)
Season of blood collection	50		97			
Rainy (Sep–Oct)		17 (34.0%)		56 (57.7%)	-	1
Winter (Nov–Jan)		33 (66.0%)		41 (42.3%)	0.01	2.65 (1.30, 5.40)
Fitzpatrick grade (skin)	50					
2		0 (0%)		1 (1%)	1.00	0.00 (0.00,-)
3		13 (26%)		13 (13.4%)	0.20	2.13 (0.68, 6.64)
4		29 (58%)		66 (68%)	0.89	0.93 (0.36, 2.41)
5		8 (16%)		17 (17.5%)	-	1
Gestational age at delivery <37 weeks	50	2 (4%)	97	10 (10.3%)	0.20	0.36 (0.08, 1.72)
Primary caesarean section	42	15 (35.7%)	83	19 (22.9%)	0.13	1.87 (0.83, 4.22)
Maternal complications	50	10 (20.0%)	97	13 (13.4%)	0.30	1.62 (0.65, 4.00)
Child birth weight (g)	50	3,124±491	97	3,066±439	0.46	1.00 (1.00, 1.00)
Child crown–heel length (cm)	50	49.6±2.8	97	49.4±2.3	0.58	1.04 (0.90, 1.20)
Child head circumference (cm)	50	33.4±1.6	97	33.1±1.4	0.25	1.15 (0.91, 1.45)
Child complications in the first 30 days	50	17 (34.0%)	97	35 (36.1%)	0.80	0.91 (0.45, 1.87)
Corrected calcium (mmol/L)	48	2.30±0.10	97	2.28±0.26	0.55	1.84 (0.25, 13.48)
Phosphate (mmol/L)	50	1.20±0.16	97	1.22±0.19	0.62	0.62 (0.09, 4.22)
Magnesium (mmol/L)	48	0.76±0.14	97	0.73±0.08	0.18	13.21 (0.30, 580.97)
Intact parathyroid hormone(pmol/L)	50	3.70±1.65	97	2.94±1.01	<0.01	1.60 (1.19, 2.15)
Albumin (g/L)	50	35.14±2.07	97	35.07±2.29	0.85	1.02 (0.87, 1.19)

^a^Logistic regression was used for all comparisons between pregnant women with and without vitamin D deficiency (25-hydroxyvitamin D <50 nmol/L *vs* ≥50 nmol/L).

**Table 3:** Multivariate analysis of factors associated with a plasma level of 25-hydroxyvitamin D <50 nmol/L (n = 147).

	β	Adjusted OR^a^ (95% CI)	*P*-value
Qualification, associate degree or higher	0.55	1.74 (0.69, 4.37)	0.24
Household income			
≤20000 THB	-	1	-
>20000 THB	0.31	1.36 (0.57, 3.27)	0.49
Pre-pregnancy BMI (kg/m^2^)	–0.13	0.88 (0.80, 0.97)	0.01
Season of blood collection			
Rainy (Sep–Oct)	-	1	-
Winter (Nov–Jan)	0.96	2.62 (1.18, 5.85)	0.02
Primary caesarean section	0.90	2.45 (0.95, 6.29)	0.06
Magnesium (mmol/L)	2.43	11.33 (0.27, 474.51)	0.20
Intact parathyroid hormone (pmol/L)	0.27	1.32 (0.94, 1.85)	0.11

^a^ Multiple logistic regression was used to analyse factors associated with vitamin D deficiency by exploring variables with *P* <0.2 without multicollinearity from univariate analysis.

## Discussion

The present study showed a high prevalence of deficiency and insufficiency of vitamin D in pregnant women at delivery. A total of 41.5% of these pregnant women had vitamin D insufficiency and 34% had vitamin D deficiency. Hypovitaminosis D in pregnant women has been reported in several studies worldwide to be 1–96%[33, 50–54]. Few studies had been conducted in tropical countries. In a study from Tanzania (n = 25), the prevalence of vitamin D deficiency [25(OH)D <50 nmol/L] at delivery was 0% with a mean concentration of 25(OH)D of 135.9±31.8 nmol/L[55]. A study from Vietnam (n = 64) showed that, in women in the early stages of pregnancy who had a 25(OH)D level of <50 nmol/L, the prevalence of vitamin D deficiency was 19% with a geometric mean (95% CI) of 75 (68–83) nmol/L[37]. Another study from Mysore (n = 559) reported the prevalence of vitamin D deficiency at 30-week gestation to be 66% with a median (IQR) maternal serum concentration of 25(OH)D of 37.8 (24.0–58.5) nmol/L[48]. A recent study from Mumbai (n = 150) reported the prevalence of vitamin D deficiency at 32–36 weeks of gestation to be 94% with a geometric mean (95% CI) of 26.5 (25.0–28.3) nmol/L[56].

Our study showed a higher prevalence of vitamin D deficiency with a lower mean concentration of 25(OH)D when compared with a recent prospective study from Pathum Thani in Thailand (latitude of 14.01N). In the latter (n = 120), the prevalence of vitamin D deficiency [25(OH)D <50 nmol/L] was 26.7%, 1.8% and 2.8% in first, second and third trimesters, respectively. The mean concentration of 25(OH)D was 61.4±16.6, 84.4±20.4 and 90.0±22.3 nmol/L in first, second and third trimesters, respectively[38].

In our study, education level and household income were associated with vitamin D status in pregnant women. Studies in Belgium[29], China[30], the Netherlands[57], the USA[58] and Spain[59] reported low-educated women to have a lower level of vitamin D compared with that of high-educated women because use of dietary supplements during pregnancy is associated with education level[60]. In the present study, pregnant women with a higher level of education and higher household income had a lower concentration of 25(OH)D and higher prevalence of vitamin D deficiency than pregnant women in the low-education and low-household-income group. Sources of vitamin D in humans arise from sunlight exposure as well as in the diet and dietary supplements. Skin exposure to sunlight is the major source of vitamin D. Therefore, vitamin D deficiency can be prevented by having sufficient exposure to sunlight[29, 30, 61]. Women of high socioeconomic status usually work indoors, so they spend a shorter time outdoors than those with low socioeconomic status. Additionally, vitamin D supplementation in pregnancy is not endorsed in Thailand, so the proportion of women receiving vitamin D supplementation in high-education/low-education and high-household-income/low-household-income groups was not different.

The efficacy of the synthesis of vitamin D by the skin is determined by age, skin pigmentation, and extent of body-attainable ultraviolet B (UVB) rays. The main factors that influence the magnitude of UVB rays on the earth surface are geographic latitude, season, and time of day[62].

We found a higher prevalence of vitamin D deficiency in the winter compared with the rainy season. In contrast, studies conducted in healthy adults, and in pregnant women at various gestational ages, found a higher prevalence of vitamin D deficiency in the rainy season than in the winter[38, 63]. Seasonal variation of vitamin D status is likely to be determined by the latitude and climate. In countries at higher latitudes, levels of solar UVB rays in the winter are too low to produce vitamin D because: (i) lower angles of solar radiation mean that UVB rays travel a greater distance through the atmosphere, and (ii) cloud cover can result in greater atmospheric absorption of UV radiation. Additionally, low temperature and low relative humidity have also been found to be important risk factors[64, 65]. A study of solar erythemal ultraviolet radiation in Thailand also found variations among different months of the year. In Nakhon Pathom (latitude 13.82N), which is located near Bangkok, UV irradiances are highest in March or April, stable from May to September, decline from October to December, and then increase during January to April[66].

Studies in postmenopausal women in Belgium, healthy women in Iran, and pregnant women in China revealed that residents living in areas of high levels of air pollution had a significantly increased probability of vitamin D insufficiency [30, 62, 67, 68]. This phenomenon may be because high levels of pollution can reduce ground levels of UVB rays significantly.

In Thailand, concentrations of air pollutants are higher in winter than those during the summer and rainy seasons. The major sources of air pollution in Bangkok (which is considered to be the most polluted area in Thailand) are automobile and industrial sources[69, 70]. This seasonal variation may result from meteorological dispersion such as the extremely stable low wind speeds or a weak influence from the high-pressure ridge that can reduce pollutant dispersion and cause a lack of rain scavenging during the winter[70].

We included both pre-pregnancy BMI and current BMI in the analysis because pre-pregnancy BMI has long been used as an important variable by literatures to predict many pregnancy-related conditions[71–73]. Pre-pregnancy BMI reflects the individual’s nutritional status prior to getting pregnant. During pregnancy course, BMI can be altered by many factors that are not necessarily indicative to the nutritional status of the woman, such as gestational edema, weight of feto-placental unit, etc. We found that a lower pre-pregnancy body weight and BMI were correlated with vitamin D deficiency, but not the BMI, at the time of delivery. Our findings are in contrast with a study showing a lower mean concentration of 25(OH)D in obese pregnant women compared with normal-weight women[74]. However, we measured the 25(OH)D concentration at the time of delivery but not before or in early pregnancy. Hemodilution and weight change during pregnancy can affect the 25(OH)D level[75]. We found that vitamin D-deficient women had a lower pre-pregnancy weight and BMI but gained more weight during pregnancy compared with women whose 25(OH)D concentration was ≥50 nmol/L. This higher weight gain may contribute to lower circulating levels of 25(OH)D.

Differences in plasma levels of calcium and phosphate were not found between women who were and were not deficient in vitamin D. The concentration of iPTH was significantly higher in women with vitamin D deficiency and the iPTH level showed a negative correlation with 25(OH)D concentration, a finding that was in accordance with other studies[76, 77]. Only 4% of women with vitamin D deficiency had an elevated level of iPTH. This phenomenon may have been because the iPTH response to a low level of 25(OH)D was reduced by higher levels of calcium in women without secondary hyperparathyroidism[76]. Levels of iPTH are regulated mainly by levels of ionized calcium, and not by 25(OH)D levels[78]. The total serum calcium decreases in late pregnancy due to dilutional hypoalbuminemia, but there was no significant difference in both serum ionized calcium and corrected calcium during pregnancy[79]. Nonetheless, ionized calcium or albumin-corrected serum calcium should be measured for accurate level of calcium during the pregnant state[80].

Our study had some limitations. Its cross-sectional design hampered determination of the cause–effect relationship. We did not have information regarding vitamin D levels throughout the year. Data regarding exposure to sunlight were self-reported; such data could be inaccurate and a recall bias was possible. We did not document the food sources of vitamin D because the study was conducted in a very busy labour ward, so taking a detailed history of dietary content was not feasible. Additionally, data on the vitamin D content of Thai food are limited[81].

## Conclusions

Despite year-round available sunshine, hypovitaminosis D has been reported in pregnant Thai women. Vitamin D deficiency increased in women who had a lower pre-pregnancy BMI and whose blood was collected in the winter. To obtain enough vitamin D, the daily intake of vitamin D should be increased, especially in pre-pregnancy women with a low BMI. Further studies are needed to explore the long-term impact of maternal vitamin D deficiency and the benefit of vitamin D supplementation in antenatal care programs in Thailand.
